# Supplementary material for: An Analytical Model of Sorption-Induced Static Mode Nanomechanical Sensing for Multicomponent Analytes
Source: Anal Chem. 2025 Aug 27;97(35):19306–12. doi: 10.1021/acs.analchem.5c03397 (PMC12424021; doi:10.1021/acs.analchem.5c03397)
Supplement: Supplementary file 1 [file ac5c03397_si_001.pdf]

Supporting Information

# An Analytical Model of Sorption-Induced Static Mode Nanomechanical Sensing for Multi-Component Analytes

Kosuke Minami*,†,‡ and Genki Yoshikawa†,§

† Research Center for Macromolecules and Biomaterials, National Institute for Materials Science (NIMS), 1-1 Namiki, Tsukuba, Ibaraki 305-0044 Japan
‡ International Center for Young Scientists (ICYS), National Institute for Materials Science (NIMS), 1-1 Namiki, Tsukuba, Ibaraki 305-0044 Japan
§ Materials Science and Engineering, Graduate School of Pure and Applied Science, University of Tsukuba, 1-1-1 Tennodai, Tsukuba, Ibaraki 305-8571, Japan
* Correspondence and requests for materials should be addressed to K.M.

## Contents

**SUPPORTING TEXTS** ..... **S2**

**BACKGROUND THEORY** ..... **S2**

SORPTION-INDUCED NANOMECHANICAL SENSORS. ..... S2

**DETAILED DERIVATIONS** ..... **S3**

RECURRENCE RELATIONS OF CONCENTRATIONS. ..... S3

RECURRENCE RELATIONS OF STRESSES. ..... S4

FIXED DURATION. ..... S5

**SUPPORTING FIGURES** ..... **S6**

FIGURE S1. A SCHEMATIC ILLUSTRATION OF THE MEASUREMENT SETUP FOR VAPOR MIXTURES AND SENSING. ..... S6

FIGURE S2. TYPICAL GEOMETRIES OF A CANTILEVER-TYPE NANOMECHANICAL SENSOR. ..... S6

FIGURE S3. NUMERICALLY CALCULATED RESPONSES OF BINARY MIXTURES DURING ABSORPTION OF ANALYTES. ..... S7

FIGURE S4. NUMERICALLY CALCULATED RESPONSES OF TERNARY MIXTURES DURING ABSORPTION OF ANALYTES. ..... S8

FIGURE S5. NUMERICALLY CALCULATED RESPONSES OF BINARY MIXTURES FOR MULTISTEP INJECTION-PURGE. ..... S9

**SUPPORTING TABLES** ..... **S10**

TABLE S1. CONDITIONS OF MFCS FOR PREPARING BINARY MIXTURES. ..... S10

TABLE S2. CONDITIONS OF MFCS FOR PREPARING TERNARY MIXTURES. ..... S10

**SUPPORTING REFERENCES** ..... **S11**

## Supporting Texts

### Background Theory

**Sorption-induced nanomechanical sensors.** A receptor material expands by sorption of analytes. However, it is attached to a substrate of nanomechanical sensors and is not free to expand.[S1,S2] In the case of static mode operation of a cantilever plate (Figure S2), the sorption-induced expansion causes the cantilever to bend.[S3] Several analytical solutions have been proposed for the theoretical formulation of the static mode operation of nanomechanical microcantilevers based on bi-material plate theory.[S4–S6] For example, the deflection of a free-end of a microcantilever $\Delta z$ induced by isotropic internal strain $\varepsilon_f$ in the receptor material is given by[S6]

$$\Delta z = \frac{3l^2(h_f + h_s)}{(A+4)h_f^2 + \left(A^{-1}+4\right)h_s^2 + 6h_f h_s}\varepsilon_f \tag{S1}$$

with

$$A = \frac{M_f w_f h_f}{M_s w_s h_s}, \tag{S2}$$

where the subscripts "$f$" and "$s$" denote the coating film (i.e., the receptor material) and the cantilever substrate, respectively, $l$, $h$, $w$, and $M$ represent the length, height, width, and biaxial modulus, respectively.[S2] According to the bi-material plate theory, the cantilever plate without a coating film does not deform. As confirmed, nanomechanical sensors without any coating film (in this case MSS) show negligible signal responses.[S7]

## Detailed Derivations

**Recurrence relations of concentrations.** From eq 4 in the main text with eq 3 in the main text, the recurrence relation between the 2*m*-th and 2(*m*+1)-th purge processes and that between (2*m*+1)-th injection and 2*m*-th purge processes can be found by

$$\mathbf{C}_{2(m+1)} - \mathbf{C}_{2m} = \mathbf{K}_p\left[e^{-(t-t_{2m+1})\mathbf{T}_s^{-1}} - e^{-(t-t_{2m})\mathbf{T}_s^{-1}}\right]\mathbf{C}_g, \tag{S3}$$

and

$$\mathbf{C}_{2m+1} - \mathbf{C}_{2m} = \mathbf{K}_p\left[\mathbf{I}_i - e^{-(t-t_{2m})\mathbf{T}_s^{-1}}\right]\mathbf{C}_g, \tag{S4}$$

respectively, where $m \in \mathbb{N}$. The concentrations at the first injection and purge processes (i.e., $n = 1$ and 2) can be solved by substituting eq 3 into eq 4 as

$$\mathbf{C}_{n=1} = \mathbf{K}_p\left[\mathbf{I}_i - e^{-(t-t_0)\mathbf{T}_s^{-1}}\right]\mathbf{C}_g, \tag{S5}$$

and

$$\mathbf{C}_{n=2} = \mathbf{K}_p\left[e^{-(t-t_1)\mathbf{T}_s^{-1}} - e^{-(t-t_0)\mathbf{T}_s^{-1}}\right]\mathbf{C}_g, \tag{S6}$$

respectively. Then, the recurrence relations in eqs S3 and S4 with eqs S5 and S6 can be solved and hence the concentrations at the (2*m*–1)-th and 2*m*)-th steps are given by

$$\mathbf{C}_{2m-1}(t) = \mathbf{K}_p\left[\mathbf{I}_i - \sum_{j=0}^{2(m-1)} (-1)^j e^{-(t-t_j)\mathbf{T}_s^{-1}}\right]\mathbf{C}_g, \tag{S7}$$

and

$$\mathbf{C}_{2m}(t) = \mathbf{K}_p\left[- \sum_{j=0}^{2(m-1)} (-1)^j e^{-(t-t_j)\mathbf{T}_s^{-1}}\right]\mathbf{C}_g, \tag{S8}$$

respectively. Then, eqs S5 and S6 can be simplified by using the indicator function $\mathbf{1}_A(n)$ (see main text) as

$$\mathbf{C}_n(t) = \mathbf{K}_p\left[\mathbf{1}_A\mathbf{I}_i - \mathbf{A}_n\right]\mathbf{C}_g. \tag{S9}$$

**Recurrence relations of stresses.** By substituting eqs 2 and 5 into eq 1, the recurrence relation between the 2*m*-th and 2(*m*+1)-th purge processes and that between (2*m*+1)-th injection and 2*m*-th purge processes can be found by

$$\sigma_{2(m+1)} - \sigma_{2m} = M_\infty \mathbf{\Lambda K}_p \left\{ \mathbf{B}\left[e^{-\left(t-t_{2m+1}\right)\mathbf{T}_s^{-1}} - e^{-(t-t_{2m})\mathbf{T}_s^{-1}}\right] + (\mathbf{I}_i - \mathbf{B})\left(e^{-\frac{t-t_{2m+1}}{\tau_r}} - e^{-\frac{t-t_{2m}}{\tau_r}}\right)\right\} \mathbf{C}_g, \quad \text{(S10)}$$

and

$$\sigma_{2m+1} - \sigma_{2m} = M_\infty \mathbf{\Lambda K}_p \left[\mathbf{I}_i - \mathbf{B}e^{-(t-t_{2m})\mathbf{T}_s^{-1}} - (\mathbf{I}_i - \mathbf{B})e^{-\frac{t-t_{2m}}{\tau_r}}\right] \mathbf{C}_g, \quad \text{(S11)}$$

respectively. The stresses at the first injection and purge processes (i.e., $n$ = 1 and 2) can be solved by substituting eqs 2 and 5 into eq 1 as

$$\sigma_{n=1} = M_\infty \mathbf{\Lambda K}_p \left[\mathbf{I}_i - \mathbf{B}e^{-(t-t_0)\mathbf{T}_s^{-1}} - (\mathbf{I}_i - \mathbf{B})e^{-\frac{t-t_0}{\tau_r}}\right] \mathbf{C}_g, \quad \text{(S12)}$$

and

$$\sigma_{n=2} = M_\infty \mathbf{\Lambda K}_p \left\{ \mathbf{B}\left[e^{-(t-t_1)\mathbf{T}_s^{-1}} - e^{-(t-t_0)\mathbf{T}_s^{-1}}\right] + (\mathbf{I}_i - \mathbf{B})\left(e^{-\frac{t-t_1}{\tau_r}} - e^{-\frac{t-t_0}{\tau_r}}\right)\right\} \mathbf{C}_g, \quad \text{(S13)}$$

respectively. Then, the recurrence relations in eqs S10 and S11 with eqs S12 and S13 can be solved and hence the stresses at (2*m*–1)-th and 2*m*-th steps are given by

$$\sigma_{2m-1}(t) = M_\infty \mathbf{\Lambda K}_p \left[\mathbf{I}_i - \mathbf{A}_n\mathbf{B} - (\mathbf{I}_i - \mathbf{B}) \sum_{j=0}^{2(m-1)} (-1)^j e^{-\frac{t-t_j}{\tau_r}}\right] \mathbf{C}_g, \quad \text{(S14)}$$

and

$$\sigma_{2m}(t) = M_\infty \mathbf{\Lambda K}_p \left[-\mathbf{A}_n\mathbf{B} - (\mathbf{I}_i - \mathbf{B}) \sum_{j=0}^{2(m-1)} (-1)^j e^{-\frac{t-t_j}{\tau_r}}\right] \mathbf{C}_g, \quad \text{(S15)}$$

respectively. Then, eqs S14 and S15 can be simplified as

$$\sigma_n(t) = M_\infty \mathbf{\Lambda K}_p \left[\mathbf{1}_A \mathbf{I}_i - \mathbf{A}_n\mathbf{B} - a_n(\mathbf{I}_i - \mathbf{B})\right] \mathbf{C}_g. \quad \text{(S16)}$$

**Fixed Duration.** Eqs 6 and 8 can be simplified when the duration t of each injection and purge is fixed (i.e., t = $t_n - t_{n-1}$) and are given by

$$\begin{aligned}\mathbf{A}_n(t) &= e^{-(t-t_0)\mathbf{T}_s^{-1}}\left[\mathbf{I}_i - \left(-e^{\tau\mathbf{T}_s^{-1}}\right)^n\right]\left(\mathbf{I}_i + e^{\tau\mathbf{T}_s^{-1}}\right)^{-1} \\ &= e^{-(t-t_{n-1})\mathbf{T}_s^{-1}}\left[e^{-n\tau\mathbf{T}_s^{-1}} - (-1)^n\mathbf{I}_i\right]\left(\mathbf{I}_i + e^{\tau\mathbf{T}_s^{-1}}\right)^{-1},\end{aligned} \tag{S17}$$

and

$$a_n(t) = e^{-\frac{t-t_0}{\tau_r}}\frac{1-\left(-e^{\frac{\tau}{\tau_r}}\right)^n}{1+e^{\frac{\tau}{\tau_r}}} = e^{-\frac{t-t_{n-1}}{\tau_r}}\frac{e^{-\frac{n\tau}{\tau_r}} - (-1)^n}{1+e^{\frac{\tau}{\tau_r}}}, \tag{S18}$$

respectively.

## Supporting Figures

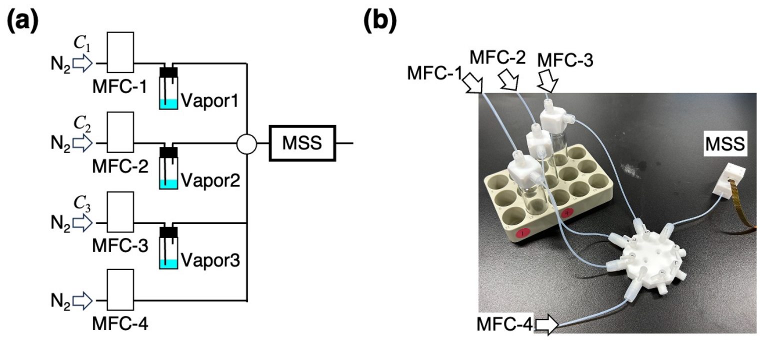


**Figure S1.** Experimental setup. **a**) A schematic illustration of the measurement setup for vapor mixtures and sensing. **b**) Photo of the part of experimental setup including three vials, mixing chamber, and MSS homemade Teflon chamber. This setup was placed in an incubator (see Experimental Section).

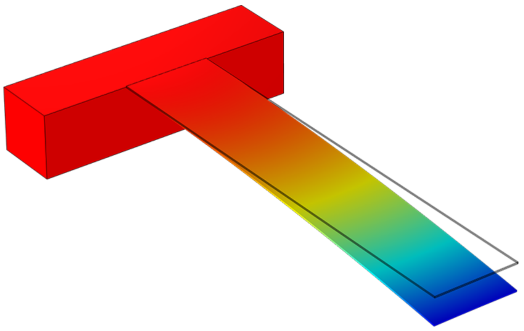

**Figure S2.** Typical geometries of a cantilever-type nanomechanical sensor. Color gradient represents the displacement in *z*-direction (i.e., perpendicular to the cantilever surface or membrane surface) simulated by finite element analysis (COMSOL Multiphysics with the Structural Mechanics module).

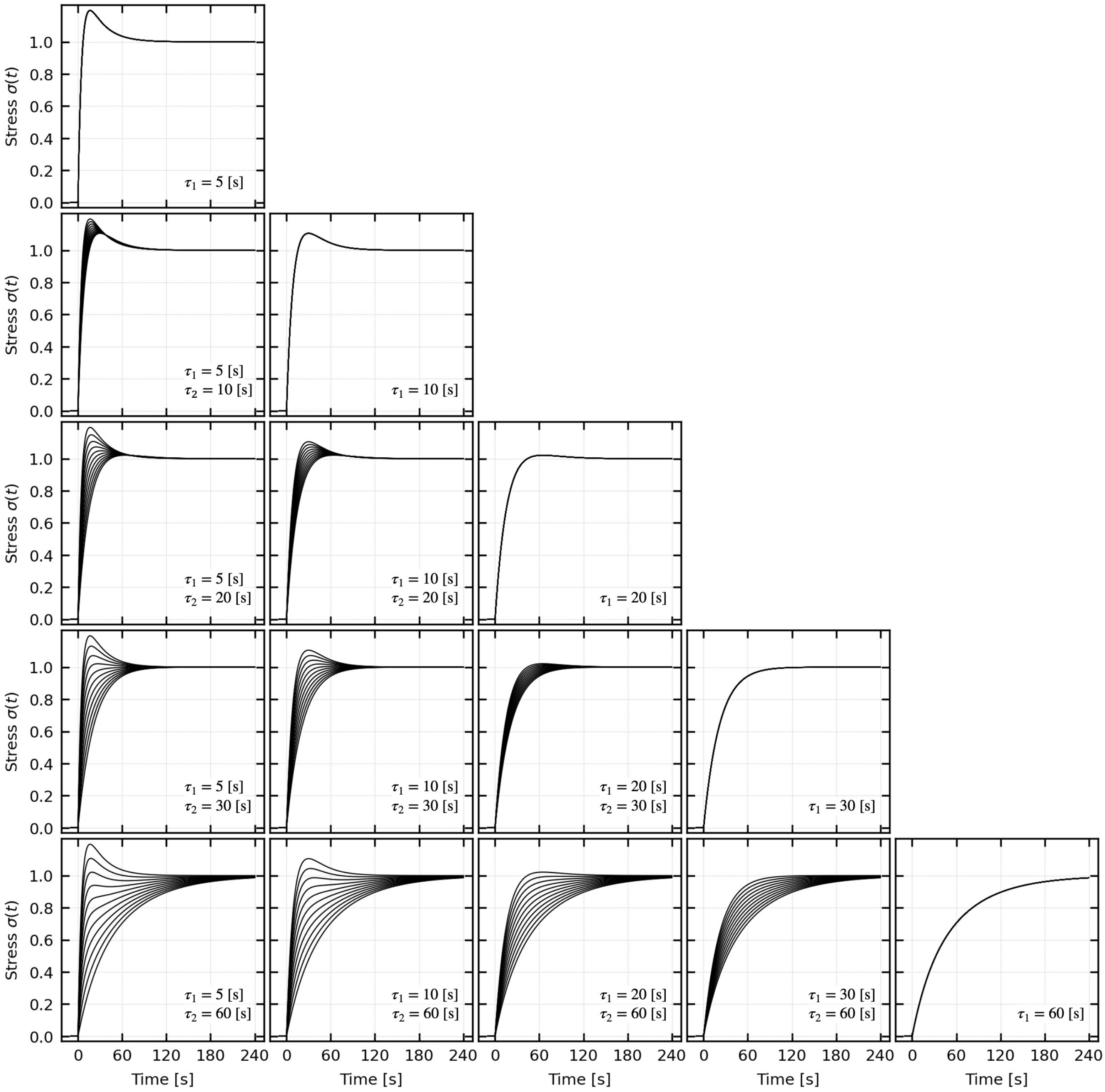


**Figure S3.** Numerically calculated responses of binary mixtures during absorption of analytes.

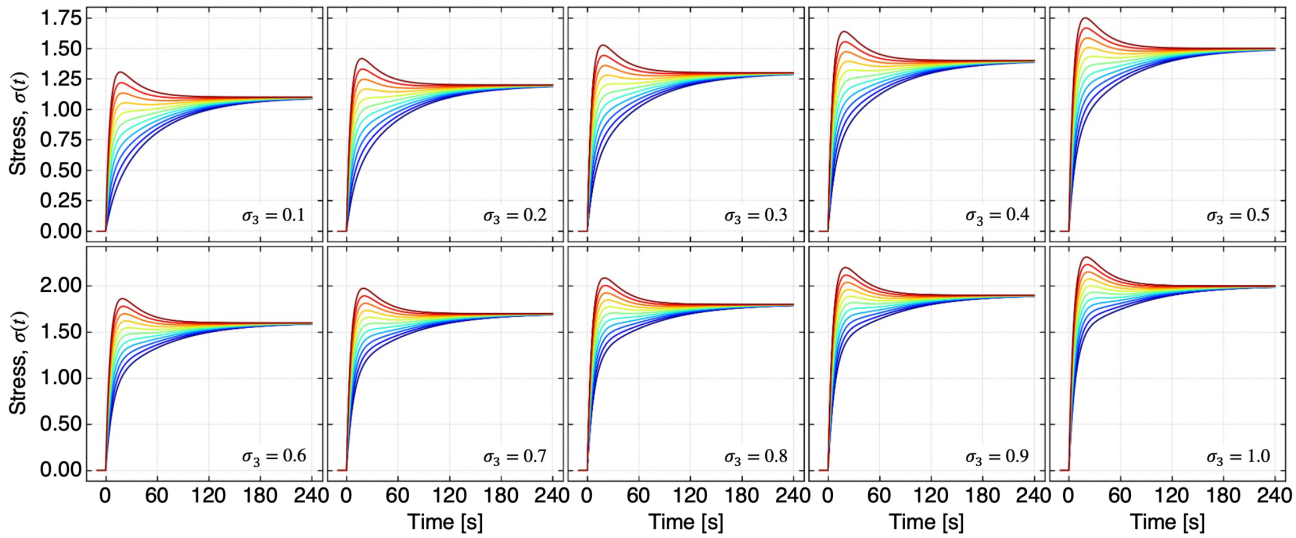


**Figure S4.** Numerically calculated responses of ternary mixtures during absorption of analytes. $\sigma_1$ = 0.0–1.0, $\sigma_1 + \sigma_2$ = 1.0, $\sigma_3$ = 0.1–1.0, $\tau_1$ = 5 [s], $\tau_2$ = 60 [s], $\tau_3$ = 8 [s].

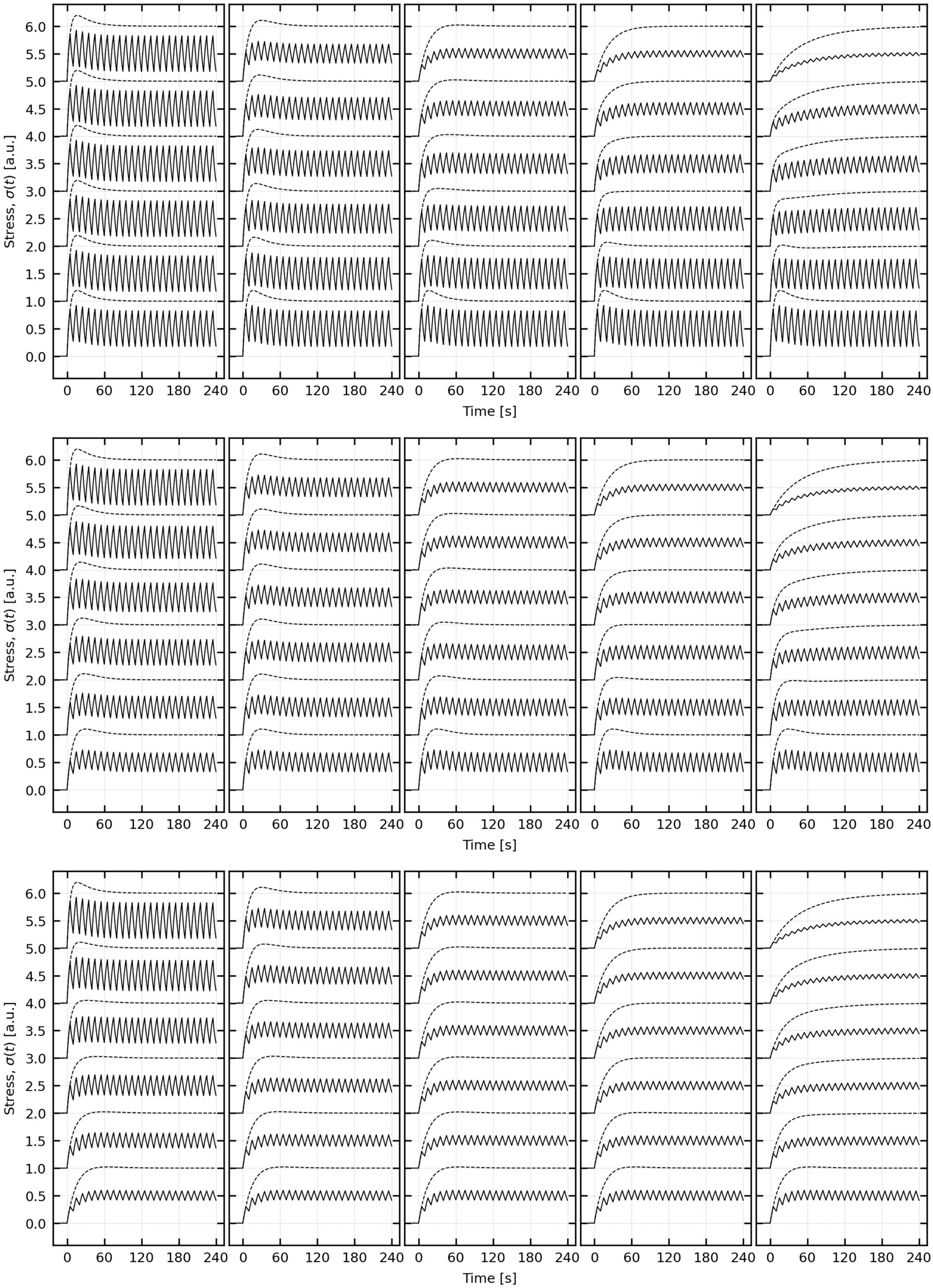


**Figure S5.** Numerically calculated responses of binary mixtures for multistep injection-purge.

## Supporting Tables

**Table S1.** Conditions of MFCs for preparing binary mixtures.

| Entry | Concentration [a] | | MFC-1 | MFC-2 | MFC-3 | MFC-4 |
|---|---|---|---|---|---|---|
| | $C_1$ | $C_2$ | [mL/min] | [mL/min] | [mL/min] | [mL/min] |
| 1 | 0% | 30% | 0 | 30 | 0 | 70 |
| 2 | 5% | 25% | 5 | 25 | 0 | 70 |
| 3 | 10% | 20% | 10 | 20 | 0 | 70 |
| 4 | 15% | 15% | 15 | 15 | 0 | 70 |
| 5 | 20% | 10% | 20 | 10 | 0 | 70 |
| 6 | 25% | 5% | 25 | 5 | 0 | 70 |
| 7 | 30% | 0% | 30 | 0 | 0 | 70 |

[a] Concentration is $P_i/P_i^o$, where $P_i$ and $P_i^o$ are the partial pressure and saturated vapor pressure of the $i$-th analyte.

**Table S2.** Conditions of MFCs for preparing ternary mixtures.

| Entry | Concentration [a] | | | MFC-1 | MFC-2 | MFC-3 | MFC-4 |
|---|---|---|---|---|---|---|---|
| | $C_1$ | $C_2$ | $C_3$ | [mL/min] | [mL/min] | [mL/min] | [mL/min] |
| 1 | 0% | 10% | 2% | 0 | 10 | 2 | 70 |
| 2 | 2% | 8% | 2% | 2 | 8 | 2 | 70 |
| 3 | 4% | 6% | 2% | 4 | 6 | 2 | 70 |
| 4 | 6% | 4% | 2% | 6 | 4 | 2 | 70 |
| 5 | 8% | 2% | 2% | 8 | 2 | 2 | 70 |
| 6 | 10% | 0% | 2% | 10 | 0 | 2 | 70 |
| 7 | 0% | 10% | 10% | 0 | 10 | 10 | 70 |
| 8 | 2% | 8% | 10% | 2 | 8 | 10 | 70 |
| 9 | 4% | 6% | 10% | 4 | 6 | 10 | 70 |
| 10 | 6% | 4% | 10% | 6 | 4 | 10 | 70 |
| 11 | 8% | 2% | 10% | 8 | 2 | 10 | 70 |
| 12 | 10% | 0% | 10% | 10 | 0 | 10 | 70 |

[a] Concentration is $P_i/P_i^o$, where $P_i$ and $P_i^o$ are the partial pressure and saturated vapor pressure of the $i$-th analyte.

## Supporting References


(S1) Wenzel, M. J.; Josse, F.; Heinrich, S. M.; Yaz, E.; Datskos, P. G., Sorption-induced static bending of microcantilevers coated with viscoelastic material. *J. Appl. Phys.* **2008**, *103,* 064913. https://doi.org/10.1063/1.2902500

(S2) Minami, K.; Shiba, K.; Yoshikawa, G., Sorption-induced static mode nanomechanical sensing with viscoelastic receptor layers for multistep injection-purge cycles. *J. Appl. Phys.* **2021**, *129,* 124503. https://doi.org/10.1063/5.0039045

(S3) Gimzewski, J. K.; Gerber, C.; Meyer, E.; Schlittler, R. R., Observation of a chemical reaction using a micromechanical sensor. *Chem. Phys. Lett.* **1994**, *217,* 589–594. https://doi.org/10.1016/0009-2614(93)e1419-h

(S4) Sader, J. E., Surface stress induced deflections of cantilever plates with applications to the atomic force microscope: Rectangular plates. *J. Appl. Phys.* **2001**, *89,* 2911–2921. https://doi.org/10.1063/1.1342018

(S5) Sader, J. E., Surface stress induced deflections of cantilever plates with applications to the atomic force microscope: V-shaped plates. *J. Appl. Phys.* **2002**, *91,* 9354–9361. https://doi.org/10.1063/1.1470240

(S6) Yoshikawa, G., Mechanical analysis and optimization of a microcantilever sensor coated with a solid receptor film. *Appl. Phys. Lett.* **2011**, *98,* 173502. https://doi.org/10.1063/1.3583451

(S7) Yeung, H. H.-M.; Yoshikawa, G.; Minami, K.; Shiba, K., Strain-based chemical sensing using metal–organic framework nanoparticles. *J. Mater. Chem. A* **2020**, *8,* 18007–18014. https://doi.org/10.1039/d0ta07248f
